# Implementation gaps in phenylketonuria management: a multicenter clinician survey from Turkiye

**DOI:** 10.1186/s13023-026-04536-7

**Published:** 2026-08-06

**Authors:** Engin Köse, Berrak Bilginer Gürbüz, Halil Tuna Akar, Tuğba Akbey Koçak, Merve Aslantaş, Esra Er, Pelin Teke Kısa, İbrahim Taş, Nazmiye Tüzel Gündüz, Merve Yoldaş Çelik, Şahin Erdöl

**Affiliations:** 1 Department of Pediatric Metabolism, Ankara University Faculty of Medicine, Ankara, Türkiye; 2 Department of Pediatric Metabolism, Yıldırım Beyazıt University, Ankara, Türkiye; 3 Department of Pediatric Metabolism, Etlik City Hospital, Ankara, Türkiye; 4 Department of Pediatric Metabolism, Mardin Training and Research Hospital, Mardin, Türkiye; 5 Department of Pediatric Metabolism, Şanlıurfa Training and Research Hospital, Şanlıurfa, Türkiye; 6 Department of Pediatric Metabolism, Dr. Behçet Uz Children’s Hospital, University of Health Sciences, İzmir, Türkiye; 7 Department of Pediatric Metabolism, Dokuz Eylül University Faculty of Medicine, İzmir, Türkiye; 8 Department of Pediatric Metabolism, Ümraniye Training and Research Hospital, İstanbul, Türkiye; 9 Department of Pediatric Metabolism, Çukurova University Faculty of Medicine, Adana, Türkiye; 10 Department of Pediatric Metabolism, Adana City Hospital, Adana, Türkiye; 11 Department of Pediatric Metabolism, Uludağ University Faculty of Medicine, Bursa, Türkiye

**Keywords:** Phenylketonuria, Rare disease, Monitoring, Dietary management, Adherence, Implementation gap

## Abstract

**Background:**

Phenylketonuria (PKU) is a rare inherited metabolic disorder requiring lifelong dietary management and regular monitoring to maintain metabolic control. Although international guidelines provide clear recommendations, their implementation in real-world clinical practice may be limited by structural and patient-related factors. This study aimed to evaluate clinician-reported PKU management practices across multiple centers in Turkiye and to identify key barriers to guideline implementation.

**Methods:**

A descriptive cross-sectional survey was conducted among metabolic specialists from multiple centers across Turkiye during a scientific meeting. The questionnaire, developed based on international PKU guidelines, assessed monitoring practices, dietary management, and barriers to implementation. Participants reported both theoretically recommended and clinician-reported monitoring frequencies across different age groups. Data were analyzed using descriptive statistics, and differences between recommended and reported practices were evaluated using the Stuart–Maxwell test.

**Results:**

Sixteen metabolic specialists from 16 centers participated. While 69% of clinicians recommended weekly monitoring in infants, only 25% reported achieving this in practice (*p* = 0.018). Similar discrepancies were observed in adolescents and adults (*p* = 0.009 and *p* = 0.043, respectively), whereas no significant difference was found in children aged 1–12 years (*p* = 0.122). Home-based dried blood spot monitoring was not routinely used in 69% of centers. The most important barriers to optimal monitoring included lack of home sampling systems, geographical distance, and work or school-related constraints. Substantial variability was also observed in dietary management practices, including protein prescription and protein substitute use.

**Conclusions:**

PKU management in Turkiye is characterized by a clear gap between guideline recommendations and clinician-reported practices, primarily driven by structural and implementation-related barriers. These findings highlight the importance of strengthening healthcare infrastructure, improving access to monitoring tools, and integrating patient-centered strategies to enhance the real-world implementation of evidence-based care in rare diseases.

**Supplementary Information:**

The online version contains supplementary material available at https://doi.org/10.1186/s13023-026-04536-7.

## Introduction

Phenylketonuria (PKU) is a rare autosomal recessive inherited disorder of phenylalanine metabolism caused by deficiency of the enzyme phenylalanine hydroxylase (PAH), which converts phenylalanine into tyrosine. Impaired PAH activity leads to elevated blood phenylalanine (Phe) concentrations that cross the blood–brain barrier and exert neurotoxic effects, resulting in intellectual disability, seizures, and behavioral disturbances if left untreated [1]. With the implementation of newborn screening and early initiation of treatment, Advances in newborn screening, dietary treatment, and pharmacological therapies have transformed PKU from a severe neurological disorder into a chronic condition in which favorable long-term outcomes are achievable. Nevertheless, lifelong management remains demanding and requires continuous dietary adherence, regular metabolic monitoring, comprehensive patient education, and sustained multidisciplinary support [2].

The prevalence of PKU varies geographically, with higher frequencies reported in certain populations, including Turkiye, where PKU represents one of the most common inherited metabolic disorders [3]. This high disease burden places a substantial responsibility on healthcare systems and metabolic centers to ensure effective long-term management.

The cornerstone of PKU treatment remains dietary phenylalanine restriction, combined with the use of phenylalanine-free or low-phenylalanine protein substitutes to ensure adequate protein intake and normal growth [4]. Achieving optimal metabolic control requires not only appropriate dietary prescription but also regular monitoring of blood Phe levels, which guides adjustments in natural protein tolerance and amino acid supplementation. In this context, treatment adherence, monitoring frequency, and individualized dietary management are key determinants of clinical outcomes [5–7].

Over the past decades, several international guidelines have been developed to standardize PKU management, including the European PKU guidelines and their recent 2025 revision, as well as recommendations from the American College of Medical Genetics and Genomics (ACMG). These guidelines provide evidence-based recommendations on target Phe levels, monitoring frequency, and dietary management strategies, aiming to optimize neurocognitive outcomes and improve consistency in care [8–11].

Despite the availability of comprehensive international guidelines, substantial variability in PKU management persists across countries and even between centers within the same healthcare system [9]. Previous studies have demonstrated significant heterogeneity in monitoring practices, dietary prescriptions, and metabolic outcomes across European centers [4, 12]. This variability is influenced by differences in healthcare infrastructure, access to specialized dietary products, reimbursement systems, patient adherence, and the practical burden of lifelong dietary treatment [9, 13]. In addition, variations in protein substitute prescription practices further highlight the lack of uniformity in clinical management [14]. Together, these factors make full adherence to guideline recommendations challenging in routine clinical practice.

In countries with a high prevalence of PKU, such as Turkiye, understanding how guidelines are translated into routine clinical practice is particularly important for identifying gaps between recommendations and implementation. However, data evaluating the real-world application of international guidelines across different centers remain limited.

Therefore, this study aimed to explore clinician-reported PKU management practices across multiple centers in Turkiye, focusing on monitoring frequency, dietary management, and adherence to guideline recommendations, and to identify key barriers contributing to inter-center variability.

## Methods

### Study design and participants

This study was designed as a cross-sectional survey evaluating clinician-reported practices in the management of phenylketonuria (PKU). The survey was conducted among metabolic specialists from multiple centers across Turkiye during a scientific meeting. It captured clinician-reported practices and perceptions rather than objective patient-level data and should be interpreted accordingly. Participation was voluntary and anonymous.

A total of 16 metabolic specialists completed the survey, representing centers providing care for both pediatric and adult PKU patients.

### Survey development and content

The questionnaire was developed based on the European PKU Guidelines (2017), the first revision of the European PKU Guidelines (2025), the evidence-based ACMG guideline for phenylalanine hydroxylase deficiency, the PKU Dietary Handbook, and previously published European studies evaluating blood phenylalanine monitoring, dietary management, and protein substitute prescription. Publications were selected based on their relevance to contemporary PKU management and their focus on key aspects of routine clinical care, including monitoring practices, dietary management, protein substitute use, sapropterin treatment, and barriers to guideline implementation. Questionnaire items were designed to reflect these core domains and to enable comparison between guideline recommendations and reported real-world clinical practice.

It consisted of five sections:

1. Center and participant characteristics.
2. Guideline use and target phenylalanine levels.
3. Monitoring frequency (theoretical vs. reported practice).
4. Protein intake and amino acid/protein substitute management.
5. Barriers to guideline implementation and areas requiring standardization.

Questions included single-choice, multiple-choice, and ranking-type items. For monitoring frequency, participants reported both theoretically recommended and reported practice frequencies across different age groups (infants, children, adolescents, and adults). The full questionnaire is provided in Supplementary File S1.

### Data collection

Data were collected using an interactive digital survey platform (Slido) during a live scientific meeting. Participation was voluntary, and responses were recorded anonymously without collection of identifiable data.

### Statistical analysis

Descriptive statistics were used to summarize the data. Categorical variables were expressed as frequencies and percentages, while continuous variables were presented as mean ± standard deviation (SD) and range (minimum–maximum), as appropriate.

For multiple-response questions, percentages were calculated based on the number of participants selecting each option.

For ranking-type questions (e.g., barriers and areas requiring standardization), a weighted scoring system was applied. Each participant selected up to three options, which were assigned weighted points (3 for the highest priority, 2 for the second, and 1 for the third). Mean scores were calculated for each option, with higher scores indicating greater perceived importance.

Differences between theoretically recommended and reported monitoring frequencies were analyzed using the Stuart–Maxwell test for marginal homogeneity. Statistical analyses were performed using IBM SPSS Statistics for Windows, Version 26.0 (IBM Corp., Armonk, NY, USA).

## Results

### Study population and practice characteristics

A total of 16 metabolic specialists from 16 centers across Turkiye participated in the survey. All centers provided care for both pediatric and adult patients with phenylketonuria (PKU). Half of the centers (50%, *n* = 8) followed more than 200 patients, while 38% (*n* = 6) managed 51–100 patients. Participants had a mean of 10.3 ± 4.4 years of experience in metabolic practice (range: 2–17 years).

Clinical decision-making for dietary management and amino acid/protein substitute prescription was predominantly multidisciplinary, with 75% (*n* = 12) reporting joint decision-making between physicians and dietitians. Only 13% (*n* = 2) reported relying exclusively on international PKU guidelines, whereas 50% (*n* = 8) primarily followed center-specific practices and 38% (*n* = 6) reported a combination of approaches.

Regarding target phenylalanine (Phe) levels, 56% (*n* = 9) supported the classical European guideline approach (≤ 360 µmol/L in childhood and ≤ 600 µmol/L thereafter), while 44% (*n* = 7) reported using a lifelong target of ≤ 360 µmol/L.

### Monitoring practices and implementation gap

**Fig. 1:**
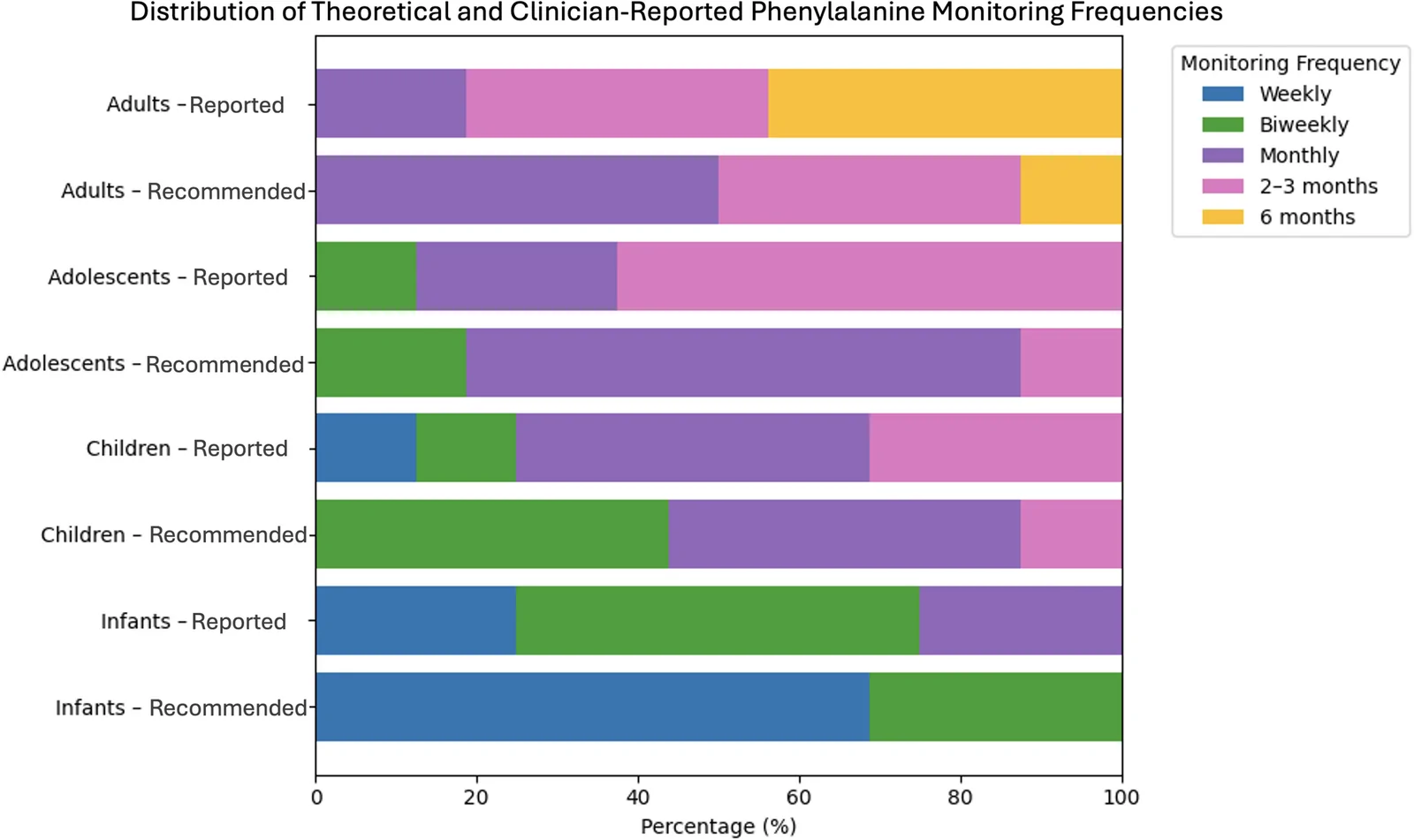
Distribution of theoretically recommended and clinician-reported phenylalanine monitoring frequencies across age groups. Reported practice shows a shift toward less frequent monitoring, particularly in adolescents and adults, with an increase in extended monitoring intervals in adult patients

A consistent discrepancy was observed between theoretically recommended and reported monitoring frequencies across all age groups (Fig. 1).

In infants (0–1 year), 69% (*n* = 11) of participants recommended weekly monitoring, whereas only 25% (*n* = 4) reported achieving this. In reported practice, monitoring was performed every two weeks in 50% (*n* = 8) and monthly in 25% (*n* = 4) (*p* = 0.018).

In children aged 1–12 years, 44% (*n* = 7) recommended monitoring every two weeks and 44% (*n* = 7) monthly. Reported practice was most commonly monthly (44%, *n* = 7) or every 2–3 months (31%, *n* = 5), with no statistically significant difference between theoretically recommended and reported frequencies (*p* = 0.122).

In adolescents, 69% (*n* = 11) recommended monthly monitoring, whereas monitoring every 2–3 months was most frequently reported (69%, *n* = 11) (*p* = 0.009).

In adults, 50% (*n* = 8) recommended monthly monitoring; however, only 19% (*n* = 3) reported achieving this. Monitoring was more commonly reported every 2–3 months (38%, *n* = 6) or every 6 months (44%, *n* = 7), indicating a shift toward less frequent monitoring (*p* = 0.043).

Participants reported increasing monitoring frequency in the presence of poor metabolic control (100%), pregnancy or preconception (100%), dietary liberalization (88%), new diagnosis (88%), and catabolic conditions such as infection (88%). Suspicion of non-adherence (81%) and initiation of sapropterin therapy (69%) were also reported.

Home-based dried blood spot (DBS) monitoring was not routinely used in the majority of centers (69%, *n* = 11), while 19% (*n* = 3) reported widespread use and 12% (*n* = 2) selective use. Although the questionnaire did not specifically evaluate alternative monitoring modalities, routine follow-up in centers not using home-based DBS generally relies on scheduled outpatient visits with conventional laboratory-based blood phenylalanine measurements.

### Dietary management and amino acid/protein substitute practices

Total protein prescription was individualized in 50% (*n* = 8) of participants, while 25% (*n* = 4) prescribed total protein intake 10–20% above the WHO/FAO/UNU safe protein intake recommendations for healthy individuals, and 25% (*n* = 4) prescribed 20–40% above these recommendations, consistent with the approach adopted in the 2025 European PKU guideline.

Protein substitutes accounted for 50–74% of total protein intake in 50% (*n* = 8) of participants, while 31% (*n* = 5) reported variability depending on individual patient characteristics. Intake was divided into three daily doses by 56% (*n* = 9) and four doses by 44% (*n* = 7).

The main determinants of amino acid dosing were blood Phe levels (100%), natural protein tolerance (94%), and age (75%). Additional factors included pregnancy (69%), growth parameters (56%), and sapropterin use (56%), whereas adherence (13%) and gastrointestinal tolerance (13%) were less frequently reported.

Use of an inefficiency factor was inconsistent, with 38% (*n* = 6) reporting routine use, 38% (*n* = 6) not using it, and the remainder applying it selectively or being unfamiliar with the concept.

All participants reported monitoring growth parameters (100%) and vitamin/mineral status (100%), while 94% (*n* = 15) monitored biochemical nutritional markers. Additional monitoring included renal function (69%), bone health (63%), and gastrointestinal symptoms (38%).

The most frequent reasons for modifying amino acid/protein substitute dosing were elevated blood Phe levels (88%), low Phe levels (71%), and growth-related changes (65%). Other contributing factors included sapropterin response (59%), increased natural protein intake (53%), and pregnancy (53%), whereas palatability or adherence-related issues (35%) and nutritional concerns (35%) were less frequently reported. Gastrointestinal side effects were uncommon (6%).

### Barriers, perceptions, and standardization needs

The main factors limiting the implementation of recommended monitoring frequency were lack of home blood sampling systems (1.88), geographical distance (1.19), and work or school-related constraints (1.00). Patient non-adherence (0.94) and financial or social factors (0.56) were also reported, while center workload (0.25) and laboratory access issues (0.13) were less frequently indicated.

**Fig. 2:**
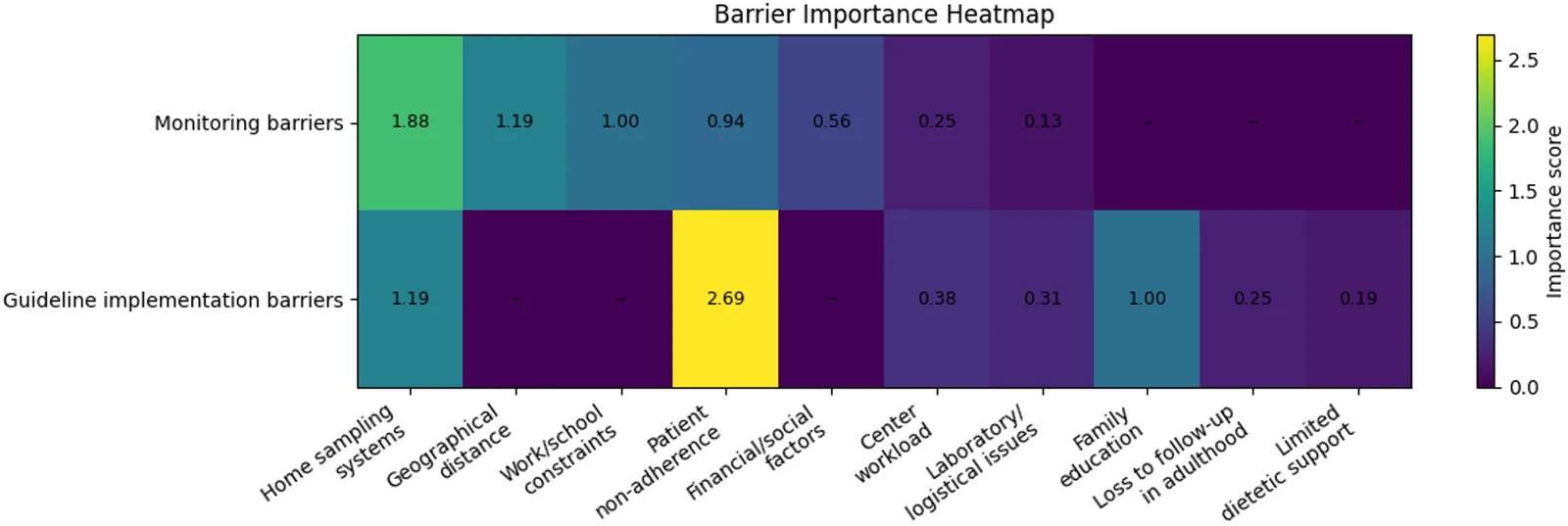
Heatmap illustrating the relative importance of barriers to monitoring frequency and overall guideline implementation in phenylketonuria management. Cell values represent weighted scores derived from ranking responses (3 = highest priority, 1 = lowest), with higher scores indicating greater perceived importance

Similarly, the most important barriers to overall guideline implementation were patient non-adherence (2.69), difficulties with home blood sampling (1.19), and insufficient family education (1.00). Other factors included center workload (0.38), laboratory and logistical constraints (0.31), loss to follow-up in adulthood (0.25), and limited dietetic support (0.19) (Fig. 2).

Areas identified as requiring standardization included monitoring frequency (2.13), adult PKU management (1.31), and sapropterin treatment algorithms (0.94), followed by target Phe levels (0.69), protein substitute dosing (0.63), and pregnancy management (0.31) (Fig. 2).

Overall, 56% (*n* = 9) of participants reported that their practice was largely consistent with guideline recommendations, while 31% (*n* = 5) reported partial consistency and 13% (*n* = 2) reported significant differences.

## Discussion

This study provides insight into clinician-reported practices and perceived gaps in PKU management in Turkiye, a country with one of the highest PKU prevalences worldwide. The most striking finding is not a lack of awareness of guideline recommendations, but rather the difficulty in consistently implementing them in routine clinical practice. Despite broad recognition of recommended monitoring strategies, a clear gap was observed between theoretical approaches and reported practice, particularly in infants, adolescents, and adults. Only a small proportion of participants relied exclusively on international guidelines, while most followed center-specific or hybrid approaches, reflecting variability in reported practice.

International guidelines emphasize that regular and sufficiently frequent blood phenylalanine monitoring is a cornerstone of optimal PKU management. The European PKU guidelines recommend weekly monitoring in infancy, every 1–2 weeks in childhood, and at least monthly monitoring in adolescents and adults, with adjustments based on metabolic stability [8, 9]. Frequent monitoring is associated with improved metabolic control, particularly in children and adolescents [12, 15]. Despite these recommendations, PKU management varies considerably across Europe, with inconsistent adherence to monitoring guidelines [4, 12]. These inter-center differences persist despite the availability of standardized recommendations, highlighting the challenges of translating guidelines into routine clinical practice [4, 16]. Consistent with these observations, our study demonstrated a significant discrepancy between theoretically recommended and reported monitoring frequencies, particularly in infants, adolescents, and adults. Although clinicians supported guideline-recommended intervals, reported practice was consistently shifted toward less frequent monitoring. This divergence was significant in infants, adolescents, and adults, but not in children aged 1–12 years, suggesting that the gap may be more pronounced at the extremes of the age spectrum.

Monitoring frequency was increased in response to clinical risk factors such as poor metabolic control and pregnancy, indicating that clinicians adapt monitoring practices dynamically despite overall constraints. Previous studies have identified multiple barriers to regular blood phenylalanine monitoring, including patient-, family-, and system-level factors. This variability is primarily driven by resource limitations, patient-related challenges, and center-specific protocols rather than a lack of guideline awareness [4, 16]. European guidelines similarly emphasize that limitations in resources, infrastructure, and implementation capacity are key obstacles to guideline adherence [9]. In addition, patient and caregiver burden such as time constraints, dietary complexity, and social limitations further complicates adherence [8]. Even in settings with home-based monitoring systems, adherence declines with age, with reduced sample return rates and worsening metabolic control [12]. Consistent with these observations, our study identified lack of home blood sampling systems, geographical distance, and work or school-related constraints as the main barriers to achieving recommended monitoring frequencies, followed by patient non-adherence and family-related factors. Importantly, patient non-adherence should not be interpreted solely as an individual patient-related issue. Lifelong dietary treatment imposes substantial practical, psychological, social, and educational demands on patients and their families. Consequently, difficulties with adherence are likely to reflect a combination of patient-, family-, and healthcare system–related factors, including access to education, availability of monitoring tools, and multidisciplinary support, rather than simple unwillingness to follow recommendations. Furthermore, because of the survey design, we were unable to distinguish whether the reported non-adherence primarily reflected insufficient patient education, limited healthcare support, psychosocial challenges, or other systemic barriers.

Home-based dried blood spot (DBS) monitoring is widely recommended to facilitate regular monitoring and reduce healthcare burden. However, effective implementation requires structured education, reliable logistics, and timely laboratory feedback [8]. Even in settings where DBS is available, adherence declines with age, particularly during adolescence and adulthood [12]. In our study, DBS was not routinely used in most centers, with 69% reporting no use and only 19% reporting widespread implementation. Lack of home sampling systems was also identified as the highest-ranked barrier, suggesting that limited access to DBS may further contribute to the gap between recommended and reported monitoring practices in Turkiye.

Previous European studies have demonstrated substantial heterogeneity in dietary management practices across centers, including differences in total protein prescription, protein substitute use, and metabolic outcomes [4, 12]. The 2025 European guideline revision recommends total protein intake at approximately 20–40% above WHO/FAO/UNU safe intake levels, while emphasizing individualized adjustment [8]. However, earlier surveys have shown that only 46% of centers apply correction or inefficiency factors, reflecting a lack of consensus [14]. Beyond documenting this variability, its potential clinical implications should be considered. When the inefficiency factor is not applied, the actual protein equivalent delivered by the substitute may be underestimated, potentially leading to suboptimal dosing despite apparently adequate prescriptions. Accordingly, the development of a consensus-based approach to the calculation and routine application of the inefficiency factor may represent an actionable priority for standardization, given its direct impact on dosing accuracy. Protein substitutes may account for approximately 52–80% of total protein intake, and dosing practices vary widely across centers [9]. Although practical guidance documents such as the PKU dietary handbook have been developed to harmonize dietary practices, variability in reported practice remains substantial [10]. Similarly, our study demonstrated substantial heterogeneity in protein and amino acid/protein substitute management. While half of participants reported individualized protein prescriptions, others used fixed approaches. The contribution of protein substitutes, dosing frequency, and the use of inefficiency factors differed across centers, indicating that variability extends beyond monitoring to dietary management and underscores the need for greater standardization.

The determinants of amino acid/protein substitute dosing in our study were predominantly biochemical, with blood phenylalanine levels and natural protein tolerance as the main factors guiding clinical decisions. In contrast, adherence-related factors were infrequently reported. Given that the effectiveness of protein substitutes depends not only on the prescribed dose but also on actual patient consumption, this finding may indicate a relative underrepresentation of adherence in dosing decisions. This observation highlights a potential gap between biochemical targets and treatment effectiveness in clinical practice. Future approaches to PKU management may benefit from greater integration of patient-reported outcomes and adherence assessment tools into routine clinical decision-making, which may represent an important area for standardization.

The increasing clinical use of sapropterin further underscores the need for standardized treatment approaches in PKU management. In our study, sapropterin use was reported as a determinant of amino acid/protein substitute dosing by 56% of participants and as a reason for dose modification by 59%, highlighting its growing relevance in clinical decision-making. Despite this, the absence of standardized protocols for dietary adjustment during the transition to sapropterin-responsive states remains a notable gap. In particular, the process of liberalizing natural protein intake and adjusting protein substitute dosing in BH4-responsive patients is not uniformly defined. These findings suggest that, alongside monitoring frequency and adult PKU management, the development of clearer and more practical sapropterin treatment approaches may represent an important priority for improving consistency in care. Importantly, successful dietary liberalization following sapropterin treatment depends not only on biochemical responsiveness but also on the patient’s lived experience. Individuals who have adhered to a low-protein diet since infancy may encounter sensory, behavioral, and psychological challenges when introducing higher-protein foods, even after treatment responsiveness has been established. Therefore, patient preferences, eating habits, and psychosocial adaptation should be considered alongside biochemical outcomes when developing practical and individualized treatment strategies.

Taken together, previous literature and the findings of the present study suggest that the existence of guidelines alone is insufficient to ensure uniform clinical practice, particularly in the presence of structural and resource-related limitations [8–10]. Consistent with this, our findings demonstrate a clear gap between guideline awareness and implementation. Although clinicians reported familiarity with international recommendations, reported practices differed substantially. Notably, more than half of participants perceived their practice as guideline-consistent despite objective discrepancies, suggesting a perception–practice gap in PKU management.

This study has several limitations. First, the sample size was relatively small (*n* = 16), and participation was limited to specialists attending a scientific meeting, which may introduce selection bias and limit generalizability. Second, data were self-reported and may not fully reflect actual clinical practice. Responses were collected in a live, interactive setting, and may therefore have been influenced by group dynamics, peer interaction, or dominant opinions rather than fully independent perspectives. In addition, the survey design did not allow differentiation between patient non-adherence resulting from insufficient education, limited healthcare support, psychosocial challenges, or other systemic factors. Therefore, the relative contribution of these factors to reported implementation barriers could not be determined. Furthermore, the survey design did not permit evaluation of the implementation of individual guideline components separately. Consequently, we were unable to determine whether more stringent recommendations, such as lower target blood phenylalanine concentrations or more frequent monitoring schedules, are implemented less consistently than more liberal recommendations. Future studies combining clinician-reported practices with objective clinical data should evaluate implementation at the level of specific guideline recommendations. Such an approach may help distinguish whether implementation barriers are predominantly related to the complexity of specific recommendations or to broader healthcare system constraints. In addition, the questionnaire was based on international guidelines but was not formally pilot-tested or validated prior to administration, which may have affected the clarity and consistency of responses. Finally, the cross-sectional design does not capture longitudinal changes, and findings from a single country may not be generalizable to other healthcare systems.

## Conclusion

In conclusion, this study highlights a clear gap between guideline-recommended and clinician-reported practices in PKU management. Our findings should be interpreted as clinician-reported perceptions rather than objective measures of care delivery. Although clinicians are generally aware of international recommendations, their implementation remains inconsistent due to structural, logistical, educational, and patient-related barriers. Improving outcomes in PKU may therefore depend less on further refining clinical guidelines and more on strengthening their implementation through enhanced healthcare infrastructure, improved access to monitoring tools, and greater integration of patient-centered approaches. Future large-scale, multicenter studies incorporating objective clinical data are needed to better characterize implementation barriers, develop standardized implementation strategies, and evaluate interventions that facilitate the translation of evidence-based recommendations into routine clinical practice. Beyond PKU, these findings may also inform implementation strategies for other inherited metabolic disorders requiring lifelong multidisciplinary care.

## Supplementary Information

Below is the link to the electronic supplementary material.

Supplementary Material 1

## Data Availability

The data that support the findings of this study are available from the corresponding author upon reasonable request. The data consist of anonymized survey responses and are not publicly available due to ethical and privacy restrictions.
