# Supplementary material for: Implementation gaps in phenylketonuria management: a multicenter clinician survey from Turkiye
Source: Orphanet J Rare Dis. 2026 Aug 6;21:395. doi: 10.1186/s13023-026-04536-7 (PMC13732954; doi:10.1186/s13023-026-04536-7)
Supplement: Supplementary file 1 [file 13023_2026_4536_MOESM1_ESM.docx]

**Survey Questionnaire**

Dear Colleagues,
Thank you for participating in this anonymous and voluntary survey evaluating clinical practices in the management of phenylketonuria (PKU), particularly regarding monitoring frequency and amino acid/protein substitute dosing.

The aim of this survey is to explore real-world clinical approaches and compare them with global PKU guideline recommendations. The findings will be used for research purposes to better understand current practices and implementation challenges.

**Section 1. Center and Participant Characteristics**

**1. Which one of the following statements applies to your center?**

*Mark only one oval.*

- We manage both pediatric and adult patients
- We only manage pediatric patients
- We only manage adult patients

**2. What is the approximate total number of PKU patients followed in your center?**

*Mark only one oval.*

- <25
- 25–50
- 51–100
- 101–200
- 200

**3. How many years have you been practicing in metabolic medicine?**

*Please specify:* __________

**4. In your center, who primarily determines dietary and amino acid/protein substitute management in PKU?**

*Mark only one oval.*

- Physician
- Dietitian
- Joint decision (physician and dietitian)
- Varies by patient

**Section 2. Guideline Use and Target Phenylalanine Levels**

**5. Which approach do you primarily follow in PKU management?**

*Mark only one oval.*

- Global PKU guidelines (European, ACMG, international consensus)
- Center-specific practice
- Combination of both
- No specific guideline

**6. What do you consider the most appropriate lifelong upper target for blood phenylalanine levels?**

*Mark only one oval.*

- ≤360 µmol/L at all ages
- ≤360 µmol/L in childhood, ≤600 µmol/L thereafter
- Depends on age and clinical context
- Unsure

**Section 3. Monitoring Frequency**

**7. In a well-controlled PKU patient aged 0–1 year, what should be the theoretical monitoring frequency?**

*Mark only one oval.*

- Weekly
- Every 2 weeks
- Monthly

**8. In practice, what is the most common monitoring frequency in this age group?**

*Mark only one oval.*

- Weekly
- Every 2 weeks
- Monthly
- Less frequent

**9. In a well-controlled PKU patient aged 1–12 years, what should be the theoretical monitoring frequency?**

*Mark only one oval.*

- Weekly
- Every 2 weeks
- Monthly
- Every 2–3 months

**10. In practice, what is the most common monitoring frequency in this age group?**

*Mark only one oval.*

- Every 2 weeks
- Monthly
- Every 2–3 months
- Less frequent

**11. In adolescents, what should be the theoretical monitoring frequency?**

*Mark only one oval.*

- Every 2 weeks
- Monthly
- Every 2–3 months
- Every 6 months

**12. In practice, what is the most common monitoring frequency in adolescents?**

*Mark only one oval.*

- Every 2 weeks
- Monthly
- Every 2–3 months
- Every 6 months

**13. In adults, what should be the theoretical monitoring frequency?**

*Mark only one oval.*

- Monthly
- Every 2–3 months
- Every 6 months
- Yearly

**14. In practice, what is the most common monitoring frequency in adults?**

*Mark only one oval.*

- Monthly
- Every 2–3 months
- Every 6 months
- Yearly

**15. Which factors make it difficult to achieve the recommended monitoring frequency?**

*Select up to 3 options.*

- Lack of home blood sampling systems
- Distance to treatment center
- Work/school constraints
- Patient non-adherence
- Family-related factors
- Financial/social barriers
- Center workload
- Laboratory access issues
- Other: ________

**16. Which conditions would lead you to increase monitoring frequency?**

*Tick all that apply.*

- Poor metabolic control
- Sapropterin initiation
- Dietary liberalization
- Pregnancy / preconception
- Growth spurt
- Suspected non-adherence
- New diagnosis
- Infection / catabolic state

**17. Is home dried blood spot (DBS) sampling routinely used in your center?**

*Mark only one oval.*

- Yes, widely used
- Yes, limited use
- No
- Only in selected patients

**Section 4. Protein Intake and Amino Acid Supplementation**

**18. How do you determine total protein requirements in PKU?**

*Mark only one oval.*

- Same as healthy population
- 10–20% higher
- 20–40% higher
- Fully individualized

**19. What proportion of total protein intake is typically provided by protein substitutes?**

*Mark only one oval.*

- <50%
- 50–74%
- 75–90%
- 90%
- Highly variable

**20. How is protein substitute intake usually divided in patients older than 1 year?**

*Mark only one oval.*

- 2 doses/day
- 3 doses/day
- 4 doses/day
- 4 doses/day

**21. Which factors are most important when adjusting amino acid/protein substitute dosing?**

*Tick all that apply.*

- Blood Phe levels
- Age
- Weight
- Growth parameters
- Natural protein tolerance
- Adherence
- Gastrointestinal tolerance
- Sapropterin use
- Pregnancy

**22. Do you use an inefficiency factor when prescribing protein substitutes?**

*Mark only one oval.*

- Yes, routinely
- Sometimes
- No
- Not familiar with this concept

**23. What parameters do you routinely monitor in patients receiving protein substitutes?**

*Tick all that apply.*

- Growth (height/weight/BMI)
- Biochemical nutritional status
- Gastrointestinal symptoms
- Renal function
- Bone health
- Vitamin/mineral status

**24. Which factors most commonly lead you to modify amino acid/protein substitute dosing?**

*Tick all that apply.*

- High blood Phe
- Low blood Phe
- Growth / weight changes
- Increased natural protein tolerance
- Sapropterin response
- Pregnancy
- Poor palatability / adherence
- Gastrointestinal side effects
- Nutritional concerns

**Section 5. Barriers and Standardization**

**25. What are the main barriers to full adherence to PKU guidelines?**

*Select up to 3 options.*

- Patient non-adherence
- Difficulty with home blood sampling
- Inadequate family education
- Center workload
- Laboratory/logistical issues
- Loss to follow-up in adulthood
- Limited dietetic support
- Product availability
- Reimbursement issues

**26. Which areas of PKU management require the most standardization in your country?**

*Select up to 3 options.*

- Monitoring frequency
- Adult PKU management
- Sapropterin treatment algorithms
- Target Phe levels
- Protein substitute dosing
- Pregnancy management

**27. Overall, how consistent is your practice with current PKU guidelines?**

*Mark only one oval.*

- Largely consistent
- Partially consistent
- Significant differences
- Unsure
